# The impact of lymphadenectomy on ovarian clear cell carcinoma: a systematic review and meta-analysis

**DOI:** 10.1186/s12957-024-03324-6

**Published:** 2024-01-29

**Authors:** Yan Liu, Feng Geng, Hongyang Zhang, Jing Xue, Ran Chu

**Affiliations:** 1 Department of Obstetrics and Gynecology, Shandong Provincial Hospital Affiliated to Shandong First Medical University, Jinan, China; 2 Department of Obstetrics and Gynecology, Qilu Hospital of Shandong University, Jinan, China

**Keywords:** Ovarian cancer, Clear cell carcinoma, Lymphadenectomy, Meta-analysis

## Abstract

**Background:**

Ovarian clear cell carcinoma (OCCC) shares treatment strategies with epithelial ovarian cancer (EOC). Due to OCCC's rarity, there's a lack of prospective studies on its surgery, resulting in heterogeneous and limited existing data. This study aims to clarify the prognostic significance of lymphadenectomy in OCCC patients.

**Methods:**

We systematically searched Web of Science, Scopus, PubMed, and Google Scholar until July 2023 for studies investigating lymphadenectomy's effects on OCCC patients. We calculated pooled hazard ratios (HR) with 95% confidence intervals (CI). This study is registered in PROSPERO (CRD42021270460).

**Results:**

Among 444 screened articles, seven studies (2883 women) met inclusion criteria. Our analysis revealed that lymphadenectomy significantly improved disease-specific survival (DSS) (HR = 0.76, 95%CI = 0.60–0.95, *P* = 0.02) and disease-free survival (DFS) (HR = 0.58, 95%CI = 0.34–0.99, *P* = 0.05). However, it did not significantly affect overall survival (OS) (HR = 0.80, 95%CI = 0.60–1.06, *P* = 0.12) or progression-free survival (PFS) (HR = 0.95, 95%CI = 0.64–1.42, *P* = 0.79). Notably, some earlier studies reported no survival benefit, warranting cautious interpretation.

**Conclusion:**

Lymphadenectomy does not significantly enhance OS and PFS for OCCC but does improve DFS and DSS. Tailoring treatment to individual patient profiles is imperative for optimal outcomes. Precise preoperative or intraoperative lymph node metastasis detection is essential for identifying candidates benefiting from lymphadenectomy. Collaborative international efforts and an OCCC database are pivotal for refining future treatment strategies.

**Supplementary Information:**

The online version contains supplementary material available at 10.1186/s12957-024-03324-6.

## Introduction

Ovarian clear cell carcinoma (OCCC), initially termed "mesonephroma" by Schiller in 1939, was believed to originate from mesonephric structures, akin to kidney cancer [1]. However, in 1973, the World Health Organization classified ovarian clear cell carcinoma as a distinct subtype within epithelial ovarian cancer (EOC) due to its unique histopathological features [2]. OCCC stands out from other histological types of EOC, exhibiting notable clinical and molecular distinctions [3–5]. Unfortunately, OCCC carries a grim prognosis and demonstrates poor responsiveness to standard chemotherapy, particularly in advanced stages [6–8].

Surgery remains the cornerstone of OCCC treatment, with surgical principles and strategies mirroring those employed for EOC. According to the current National Comprehensive Cancer Network (NCCN) guidelines [9], the standard surgical approach entails hysterectomy, bilateral salpingo-oophorectomy, omentectomy, pelvic and para-aortic lymphadenectomy, and cytoreduction surgery. It's important to note that adherence to these recommendations varies. The NCCN guidelines assign a level of evidence of category 2A to OCCC, reflecting limited clinical data available for reference. This evidence is primarily derived from a retrospective multicenter study involving 240 OCCC patients [10].

In the MITO-9 study, only 54% of early OCCC cases underwent lymphadenectomy [10]. Lymphadenectomy serves crucial roles in accurately staging the disease, providing prognostic insights, and guiding adjuvant therapy, particularly in early-stage OCCC [11]. However, considering the relatively low rate of lymph node metastases in clinically early-stage OCCC patients [12], the question arises: is it justified to perform lymphadenectomy solely for staging purposes? Hirose et al. conducted a retrospective analysis of recurrence patterns in 602 patients with stage I ovarian cancer. Among the 70 patients who experienced recurrence, 61% had peritoneal recurrences, 26% had metastasis through blood circulation, and 13% had lymphatic metastasis [13]. In the subgroup of 277 OCCC patients, 64% experienced peritoneal recurrence, 31% had blood circulation metastasis, and 15% had lymphatic metastasis. Notably, among the 226 patients who underwent systemic lymphadenectomy, only 0.9% had lymph node recurrence. In contrast, among the 376 patients who did not undergo lymphadenectomy, 1.9% experienced lymph node recurrence, with no significant difference between the two groups (*P* = 0.339). Furthermore, in light of existing retrospective literature, the effectiveness of lymphadenectomy in early OCCC patients remains a subject of controversy [14–20].

Lymphadenectomy is a common practice in the surgical treatment of advanced EOC, and OCCC is no exception [21–23]. However, a recent randomized controlled trial (RCT), the Lymphadenectomy in Ovarian Neoplasm (LION) trial, challenged this practice. It suggested that systematic lymphadenectomy may not be necessary for advanced ovarian cancer when no abnormal lymph nodes are detected in preoperative imaging and intraoperative examination, and optimal cytoreduction can be achieved. The trial found that the overall survival (OS) and progression-free survival (PFS) of patients who did not undergo lymphadenectomy were similar to those who did, with the latter group being more susceptible to surgical complications. Importantly, the study did not include patients with positive lymph nodes, and it did not conduct a subgroup analysis based on histologic subtype [24]. Additionally, in 2020, a meta-analysis of 15 studies encompassing 33,257 patients with advanced ovarian cancer who underwent lymph node resection suggested that lymphadenectomy did not confer a survival benefit in this patient population (*P* = 0.16) [25]. Most of the studies cited as evidence for standard EOC management did not include a significant number of patients with clear cell histology, making their findings less applicable to OCCC [26].

Therefore, the aim of our study was to systematically review the existing literature and conduct a meta-analysis to assess the impact of lymphadenectomy on the survival outcomes of OCCC.

## Methods

### Search strategy and selection criteria

The systematic review and meta-analysis were conducted following the Preferred Reporting Items for Systematic Reviews and Meta-Analysis (PRISMA) guidelines [27]. A PRISMA checklist is available in Supplementary material. Our comprehensive search covered Web of Science, Scopus, PubMed, and other relevant sources, such as Google Scholar, spanning from the inception of each database to July 2023. To ensure that no pertinent studies were overlooked, we manually examined the reference lists of identified articles and utilized the "Related Articles" feature in PubMed to identify additional relevant papers. Our systematic review is registered in PROSPERO under the registration number CRD42021270460. Supplementary material contains the complete search strategy for reference.

Only studies comparing survival outcomes between the lymphadenectomy and no-lymphadenectomy groups were included. Excluded were editorials, letters, case reports, single-arm studies, comments, personal communications, proceedings, non-English studies, and non-human studies, as they did not contain relevant quantitative outcomes. After removing duplicates, two independent reviewers screened titles and abstracts against the inclusion and exclusion criteria mentioned above. All remaining studies underwent full-text screening, with any disagreements between reviewers resolved through discussion to reach a consensus.

### Data extraction

Two independent reviewers extracted pertinent data from each eligible study and recorded it in a standardized data extraction form. The extracted data encompassed the first author's name, publication year, country of origin, study duration, study design, group sizes, participant ages, tumor staging, lymphadenectomy criteria, adjuvant chemotherapy details, and duration of follow-up. To obtain survival data not explicitly provided in numerical format within the articles, we utilized R-4.0.3 software to extrapolate information from Kaplan–Meier curves.

### Quality assessment

We assessed the risk of bias in the included cohort studies using the Newcastle–Ottawa scale [28], which employs a star-based system (up to a maximum of 9 stars) to evaluate studies across three domains: participant selection, comparability of study groups, and outcome ascertainment. Studies with a score of 7 stars or higher were categorized as having a low risk of bias, those with scores between 5 and 6 stars were considered to have a medium risk of bias, and studies scoring 4 stars or fewer were classified as having a high risk of bias.

### Statistical analysis

In this meta-analysis, we utilized hazard ratios (HR) obtained from time-event survival analyses to evaluate differences in disease-specific survival (DSS), disease-free survival (DFS), OS, and PFS between the lymphadenectomy and no-lymphadenectomy groups. OS is defined as the time from the start of treatment to death from any cause. PFS is defined as the time from the start of treatment to disease progression or death from any cause. DFS is the period from the start of treatment to disease recurrence or death from any cause. DSS is the period from the start of treatment to death due to a specific disease. We extracted HR values and their corresponding 95% confidence intervals (CI) directly from the original articles. In cases where this information was not available, we calculated or extrapolated the relevant results using the Parmar and Tierney methods [29, 30] based on the provided Kaplan–Meier curves.

We conducted meta-analysis using either the random-effects model or the fixed-effects model, depending on the presence or absence of significant heterogeneity among the studies. Heterogeneity assessment relied on two statistics: the Chi-squared test based on Cochran's Q-test and the I-squared statistic. If the I-squared statistic indicated substantial heterogeneity (> 50%), we employed the random-effects model, treating the studies as random samples from a hypothetical population with varying effects [31]. Study weights were determined using the inverse variance method in all cases. Pooled effects were calculated, with statistical significance set at a two-sided *P*-value of less than 0.05. Subgroup analyses were performed based on the stage of OCCC. For statistical analysis and graphical representation, we utilized R-4.0.3 software.

### Publication bias

Publication bias was evaluated using Egger’s test. The absence of publication bias was indicated by the data points forming a symmetric funnel-shaped distribution and a one-tailed significance level *P* > 0.05 (Egger’s test).

## Results

### Search results and details of included studies

**Fig. 1:**
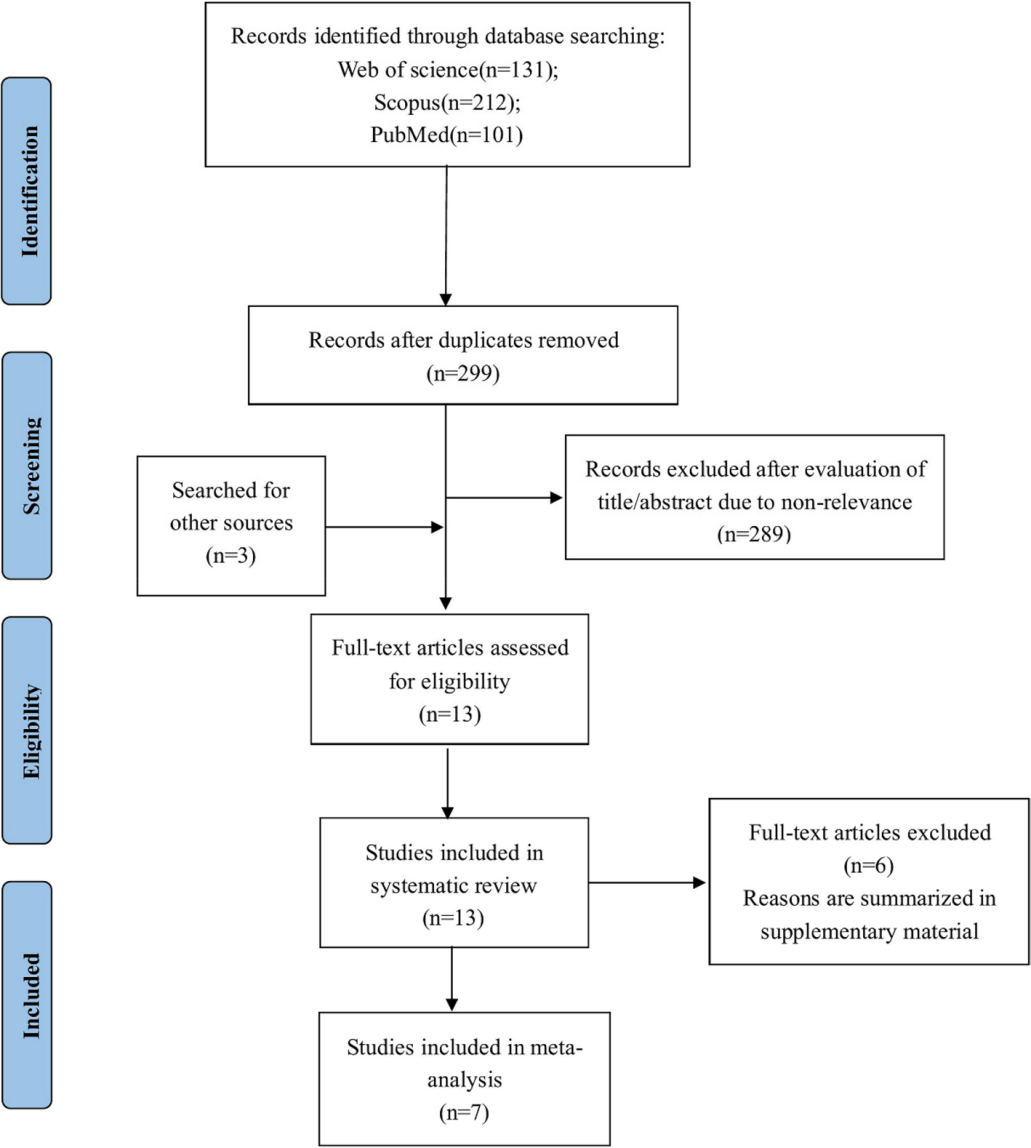
PRISMA flow diagram for study identification and inclusion

Figure 1 presents the study retrieval and selection flowchart, while reasons for full-text exclusions can be found in Supplementary material. After eliminating duplicates, our initial literature search yielded 299 articles, which underwent title and abstract review. We excluded 289 studies that were not relevant to the review topic, and an additional three potentially relevant articles were obtained through alternative sources, such as Google Scholar. Two authors independently assessed these articles for eligibility, resolving discrepancies through consensus.

Out of the 13 articles selected for full-text review, six were excluded with stated reasons, resulting in a total of seven studies meeting all inclusion criteria [10, 17–20, 32, 33]. Further details of the included studies are provided in Table 1. All of the eligible studies were retrospective observational studies. Among them, four studies [17–20] included patients with early-stage OCCC, one study [32] included patients with advanced OCCC, and two studies [10, 33] included both early and advanced cases. The lymphadenectomy group's sample sizes ranged from 52 to 1298 patients, while the no-lymphadenectomy group sizes ranged from 36 to 538. There were variations in the number of lymph nodes removed among studies, as well as differences in the factors adjusted in multivariable analysis and the duration of follow-up across studies.

**Table 1:** Basic characteristics of included studies in the meta-analysis

Author	Year	Country; Study period	Group(No.)	Definition	Age	FIGO stage (patient No)	Adjuvant chemotherapy	Follow-up (months)
Suzuki at el.	2008	Japan; 1986–2006	LA 104(50.7%)	Pelvic and para-aortic lymphadenectomy	52 (30–75)	Ia–b:19(18.3%) Ic: 76 (73.0%) IIa–b: 9(8.7%)	Platinum-based 34 (32.7%) TP 54 (51.9%) Others 1 (1.0%) None 15 (14.4%)	49.6
			No-LA 101(49.3%)	With or without sampling of lymph nodes	51 (32–75)	Ia–b:27(26.7%) Ic: 67 (66.3%) IIa–b: 7(7.0%)	Platinum-based 49 (48.5%) TP 34 (33.7%) Others 2 (1.9%) None 16 (15.8%)	49.2
Takano at el.	2009	Japan; 1992–2002	LA 124(67.3%)	Pelvic and para-aortic lymphadenectomy	NA	Ia: 32 (26%) Ic: 92 (74%)	Paclitaxel + platinum 51 (41%) Others* 64 (52%) None 9 (7%)	49 (5–130)
			No-LA 65(32.7%)	Only lymph node exploration or sampling	NA	Ia: 13 (20.0%) Ic: 52 (80.0%)	Paclitaxel + platinum 20 (31%) Others* 38 (58%) None 6 (10%)	57 (5–150)
Magazzino at el.	2011	Italy; 1991–2007	LA 115(47.9%)	Pelvic lymphadenectomy51 pelvic and para-aortic lymphadenectomy 64	56 (29–83)	I: 108 (45%) II: 30 (12.5%) III: 81 (33.7%) IV: 16 (6.6%) Not available: 5 (2.0%)	Platinum-based with taxane 127 (52.9%) Without taxane 92 (38.3%) None 21 (8.75%)	30.1 (1.4–126.6)
			No-LA 125(52.1%)	Not performed				
Mahdi at el.	2013	USA; 1988–2007	LA 1298(70.7%)	Pelvic and para-aortic lymphadenectomy	56.2	Stage I	NA	NA
			No-LA 538(29.3%)	Not performed	54.7		NA	NA
Yamazaki at el.	2018	Japan; 1995–2015	LA 91(71.7%)	Pelvic lymphadenectomy12 pelvic and para-aortic lymphadenectomy 79	53 (34–79)	Ia: 34 (26.8%) Ic: 78 (61.4%) II: 15 (11.8%)	None 34 (26.8%) Platinum-based 61 (48.0%) Nonplatinum-based 32 (25.2%)	NA
			No-LA 36(28.3%)	Not performed				NA
Kajiyama at el.	2020	Japan; 1986–2017	LA 112(67.5%)	Pelvic and para-aortic lymphadenectomy	55.0 ± 9.6	II: 53 (47.3%) III: 52 (46.4%) IV: 7 (6.3%)	None 3 (2.7%) TP 82 (73.2%) Non-TP 27 (24.1%)	54.0 (5.1– 184.2)
			No-LA 54(32.5%)	Not performed	56.5 ± 12.3	II: 25 (46.3%) III: 23 (42.6%) IV: 6 (11.1%)	None 3 (5.6%) TP 40 (74.1%) Non-TP 11 (20.5%)	50.4 (1.6–159.8)
Nasioudis at el.	2021	USA; 2010–2015	LA 52(43.3%)	Pelvic and para-aortic lymphadenectomy	NA	Stage III	NA	43.63
			No-LA 68(56.7%)	Not performed	NA		NA	53.75

*Abbreviations: LA* lymphadenectomy, *No-LA* no-lymphadenectomy, *FIGO* Federation International of Gynecology and Obstetrics, *NA* not available, *TP* taxane + platinum

### Quality assessment

All seven studies [10, 17–20, 32, 33] were observational in nature. We employed the Newcastle–Ottawa Scale to assess the quality of included cohort studies, and all seven studies received scores of 7 or higher, indicating a low risk of bias. Additional details regarding the risk of bias assessment can be found in Supplementary material.

### Meta-analysis of OS for patients with or without lymphadenectomy

**Fig. 2:**
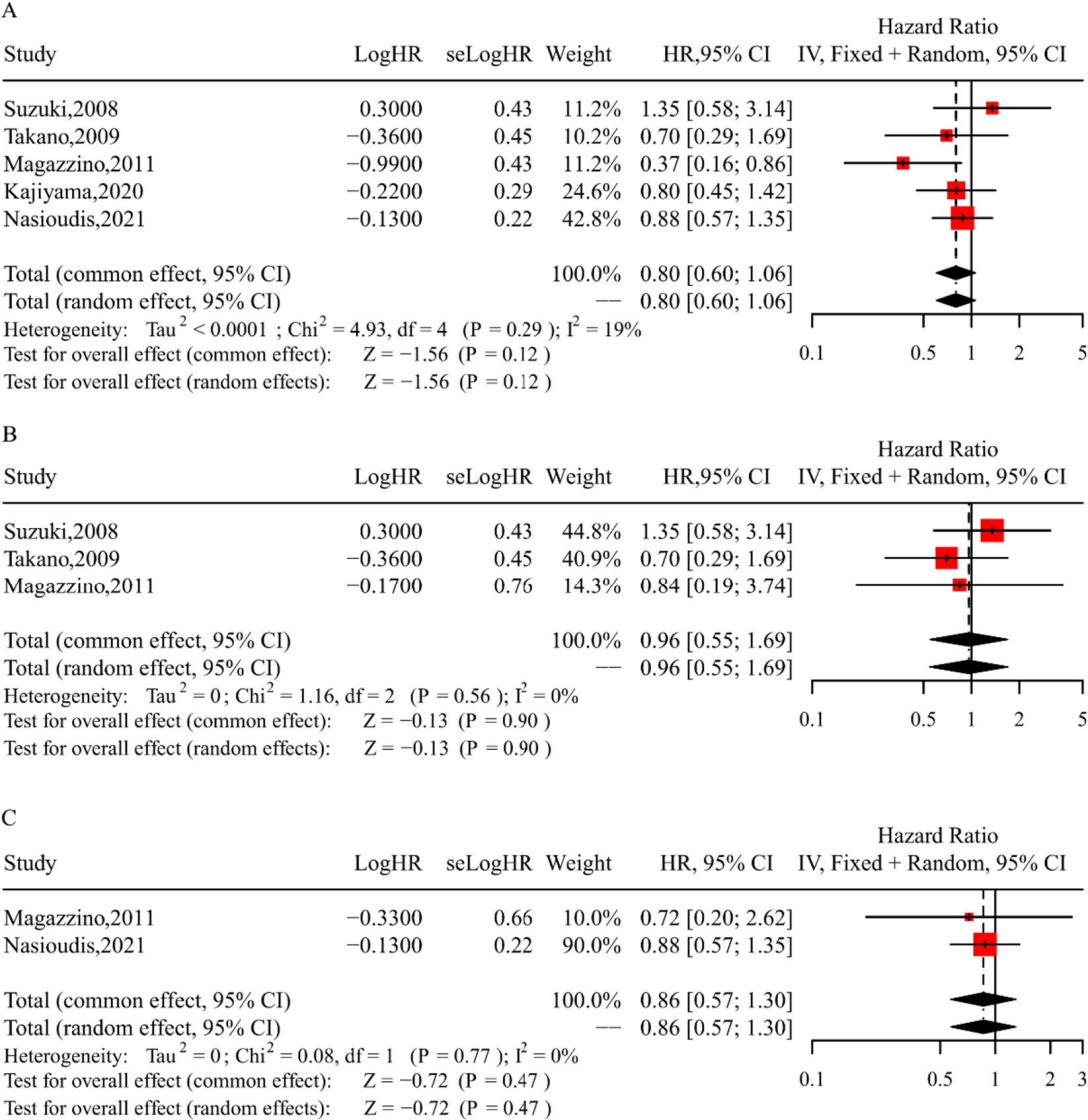
Forest plots of OS for patients with or without lymphadenectomy. **A** Forest plot of OS in all-stage for patients with or without lymphadenectomy; **B** Forest plot of OS in early-stage for patients with or without lymphadenectomy; **C** Forest plot of OS in advanced-stage for patients with or without lymphadenectomy

Five cohort studies [10, 18, 19, 32, 33] comprising a total of 920 patients were included in the analysis to calculate the pooled HR for OS between the lymphadenectomy and no-lymphadenectomy groups across all stages of the disease. A fixed-effect model was employed (Chi-square = 4.93; I^2^ = 19%; *P* = 0.29), and the analysis indicated no significant impact of lymphadenectomy on OS (HR = 0.80; 95% CI = 0.60–1.06; *P* = 0.12; Fig. 2A**)**. For the subgroup analysis of three cohort studies focused on early-stage disease, the results revealed that lymphadenectomy did not lead to an improvement in OS (HR = 0.96; 95% CI = 0.55–1.69; *P* = 0.90; Fig. 2B). Similarly, in the subgroup analysis of advanced disease, there was no significant difference in OS between the lymphadenectomy group and the no-lymphadenectomy group (HR = 0.86; 95% CI = 0.57–1.30; *P* = 0.47; Fig. 2C).

### Meta-analysis of DFS for patients with or without lymphadenectomy

**Fig. 3:**
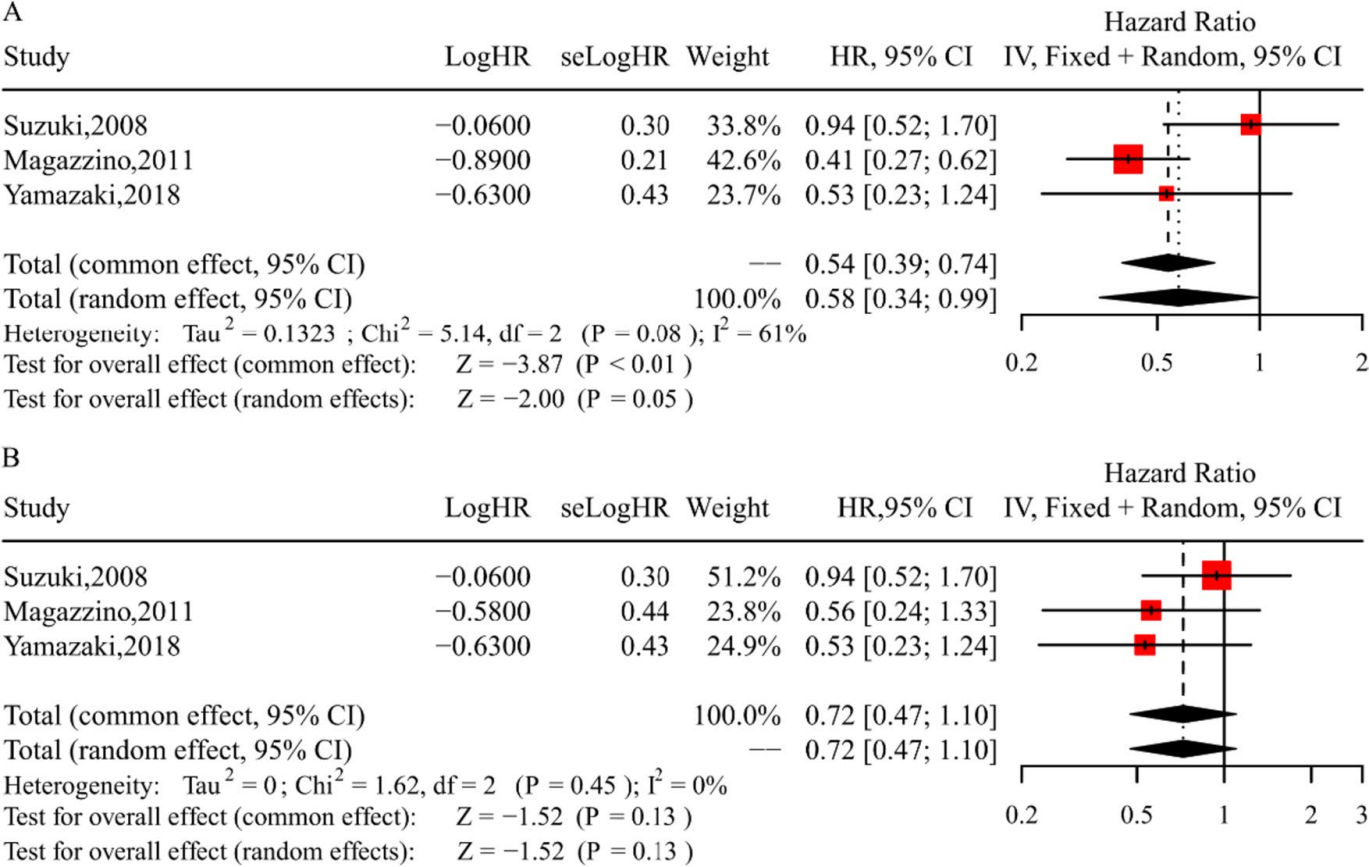
Forest plots of DFS for patients with or without lymphadenectomy. **A** Forest plot of DFS in all-stage for patients with or without lymphadenectomy; **B** Forest plot of DFS in early-stage for patients with or without lymphadenectomy

The meta-analysis, comprising three cohort studies involving 560 women, assessed disease-free survival (DFS) between the lymphadenectomy and no-lymphadenectomy groups across all stages of the disease [10, 17, 18]. We employed a random-effects model of analysis (Chi-square = 5.14; I^2^ = 61%; *P* = 0.08). The pooled analysis indicated potential benefits of lymphadenectomy for DFS when compared to the no-lymphadenectomy group (HR = 0.58; 95% CI = 0.34–0.99; *P* = 0.05; Fig. 3A). In contrast, there was no significant improvement in DFS observed for the lymphadenectomy group in early-stage OCCC (HR = 0.72; 95% CI = 0.47–1.10; *P* = 0.13; Fig. 3B).

### Meta-analysis of PFS for patients with or without lymphadenectomy

**Fig. 4:**
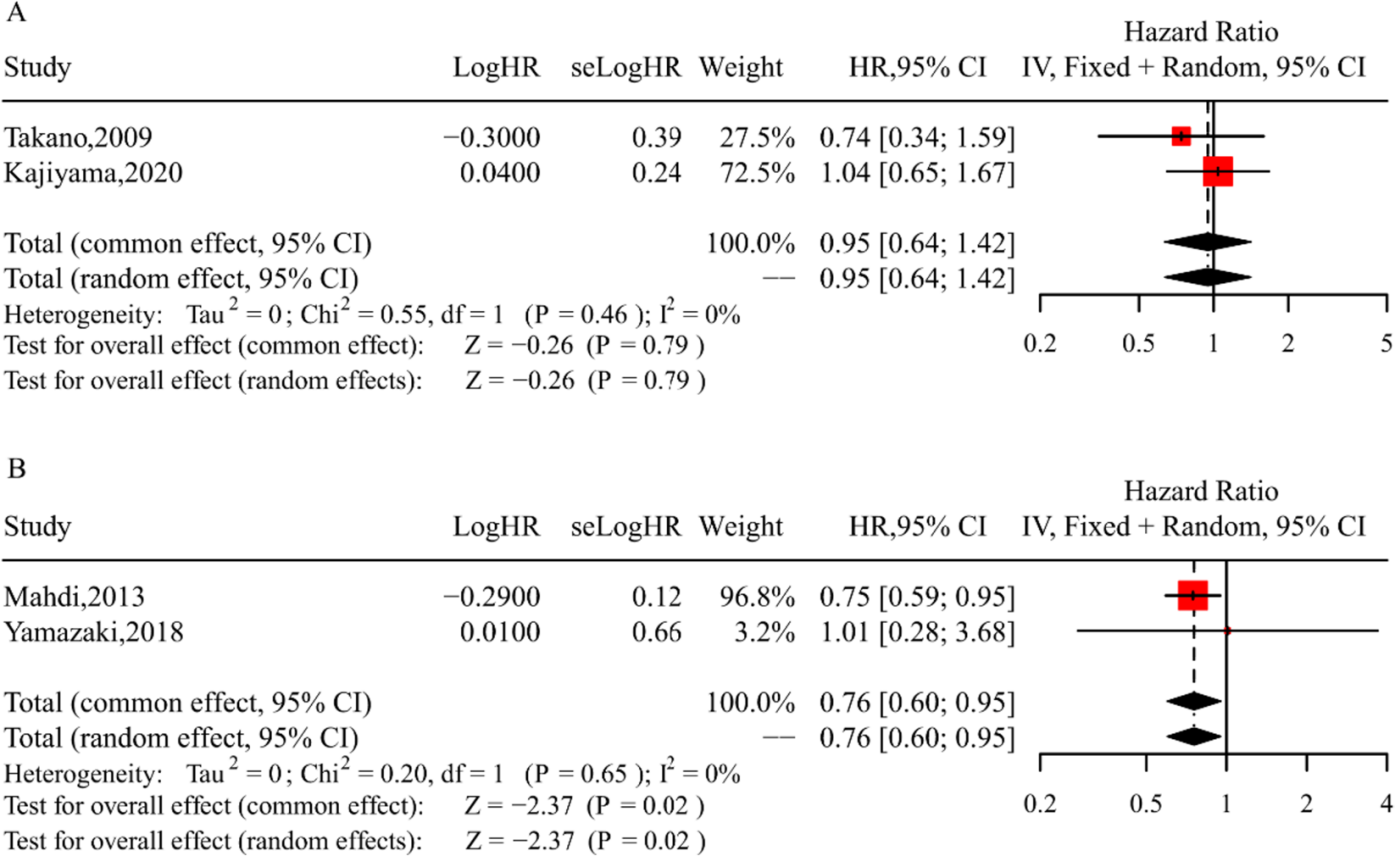
Forest plots of PFS (**A)** and DSS (**B**) for patients with or without lymphadenectomy

Two cohort studies included in our analysis provided relevant data on PFS [19, 33], encompassing a total of 355 women with all-stage disease. We utilized a fixed-effect model of analysis (Chi-square = 0.55; I^2^ = 0.0%; *P* = 0.46). The analysis did not reveal a significant difference between the lymphadenectomy and no-lymphadenectomy groups in terms of PFS (HR = 0.95; 95% CI = 0.64–1.42; *P* = 0.79; Fig. 4A).

### Meta-analysis of DSS for patients with or without lymphadenectomy

Data on DSS in early-stage patients were available from only two studies [17, 20]. The pooled HR was 0.76 (95% CI = 0.60–0.95; *P* = 0.02; Fig. 4B), indicating a statistically significant difference in DSS between the lymphadenectomy and no-lymphadenectomy groups in the meta-analysis. Heterogeneity testing did not reveal significant heterogeneity (Chi-square = 0.20; I^2^ = 0.0%; *P* = 0.65) for the DSS data.

### Publication bias

**Fig. 5:**
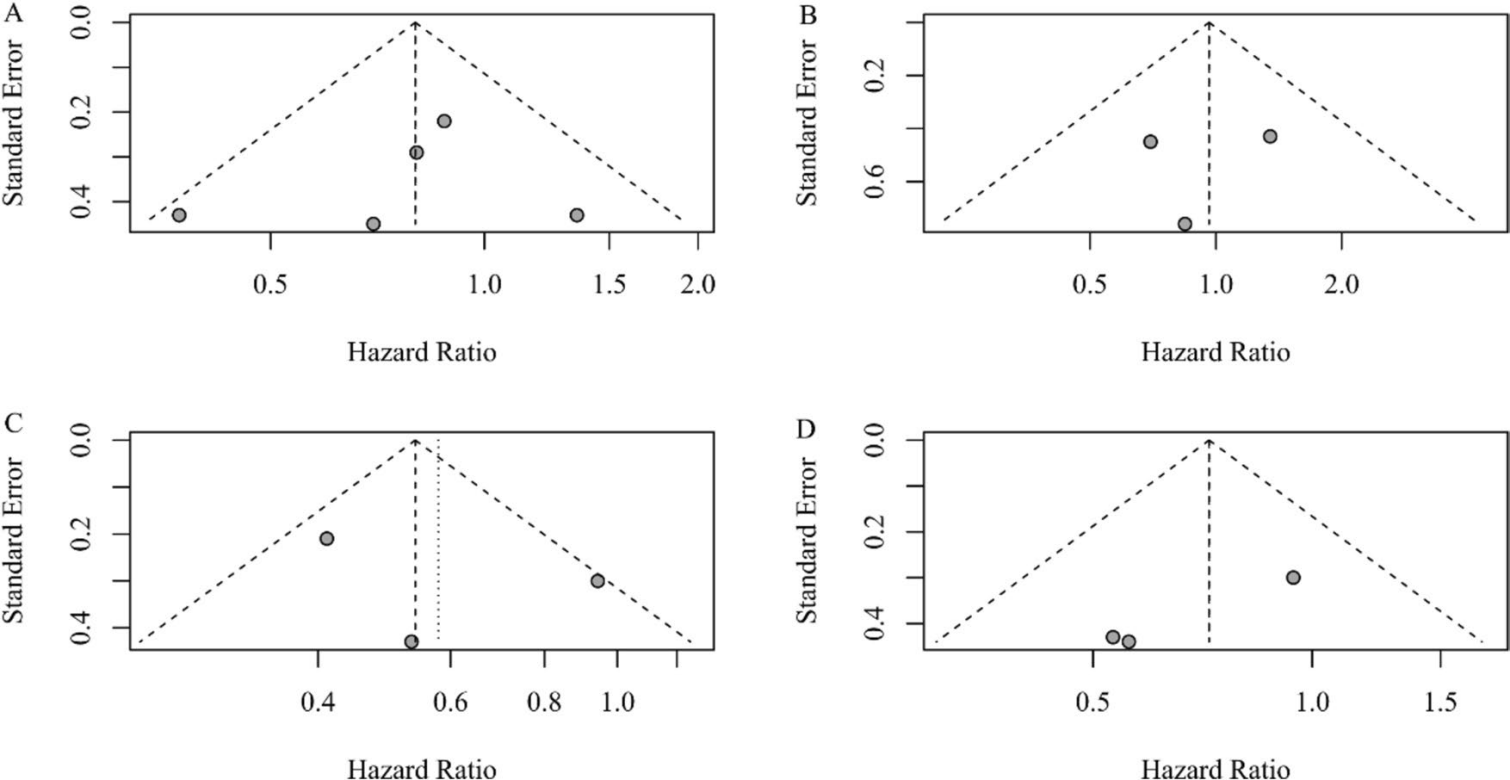
Funnel plots of standard error by hazard ratio for patients with OCCC. **A** Funnel plot of standard error by hazard ratio of OS for all-stage patients with OCCC; **B** Funnel plot of standard error by hazard ratio of OS for early-stage patients with OCCC; **C** Funnel plot of standard error by hazard ratio of DFS for all-stage patients with OCCC; **D** Funnel plot of standard error by hazard ratio of DFS for early-stage patients with OCCC

There was no evidence of significant publication bias by inspection of the formal statistical tests (Egger’s test). A detailed publication bias assessment is described in Fig. 5.

## Discussion

This meta-analysis revealed no significant differences in OS and PFS between the lymphadenectomy and no-lymphadenectomy groups. However, a potential benefit of lymphadenectomy was observed in DSS and DFS. These findings are similar to those of a recent meta-analysis conducted by Purwar et al. [34]. This meta-analysis of systematic para-aortic and pelvic lymphadenectomy in EOC showed no significant impact on survival. However, due to the histological heterogeneity of EOC, our study conducted a meta-analysis of one of these rare subspecies, which could help to provide recommendations for personalized treatment of ovarian cancer.

While lymphadenectomy for various gynecological tumors is well-established, surgical complications remain a concern. For instance, the incidence of pelvic lymphoceles after systemic para-aortic and pelvic lymphadenectomy ranges from 4.3% to 48%, often accompanied by complications such as pelvic infections, compression of adjacent tissues and organs, pain, and lower limb edema [35]. In a significant randomized controlled trial involving 427 patients with stage IIB-IV ovarian cancer, participants were randomly assigned to either systemic para-aortic and pelvic lymph node resection (216 cases) or enlarged lymph node resection only (211 cases). The study revealed a significantly higher incidence of perioperative complications (including vascular injury, small bowel obstruction, postoperative lymphoceles, deep vein thrombosis, and lower extremity edema) in the former group compared to the latter [36]. Moreover, access to surgeons experienced in lymphadenectomy and complete staging procedures may not always be readily available for the initial procedure. As a result, there remains insufficient evidence to definitively determine the influence of lymphadenectomy on the survival of patients with OCCC.

In a large retrospective study, lymph node metastasis rates for OCCC were 9.1% in pT1a and 7.1% in pT1c stages [37]. The likelihood of lymph node metastasis in early-stage OCCC appears to be relatively low, suggesting that lymphadenectomy may not be necessary in these cases. Performing lymphadenectomy only to identify positive lymph nodes in a small number of cases can result in significant overtreatment, potentially exceeding 90%. However, it's important to note that lymphadenectomy plays a crucial role in detecting metastatic lymph nodes, especially since patients with positive lymph nodes tend to have a poor prognosis. Another study indicated that lymph node metastasis rates in patients with clinical stage I OCCC, combined with positive cytology and ovarian surface involvement, reached as high as 37.5% [12]. This suggests that patients with stage I OCCC who are cytologically positive or have ovarian surface involvement require careful consideration and should not be readily exempted from lymphadenectomy.

In current clinical trials assessing lymphadenectomy for ovarian cancer, the majority of enrolled women have high-grade serous ovarian cancer, with OCCC representing a very small proportion. Consequently, it cannot be assumed that the results of these trials are applicable to other less common histologic subtypes of ovarian cancer, such as OCCC. In Chan et al.'s study examining the association of systemic lymphadenectomy with survival, patients with non-clear cell EOC experienced significantly improved disease-specific survival at 5 years after lymphadenectomy. However, the positive effects of lymphadenectomy were not statistically significant in the smaller subgroup of patients with clear cell histology [38]. This difference may be attributed to variations in sensitivity to postoperative chemotherapy or an uneven distribution of stage I tumor subtypes. In a study by Suzuki et al., it was observed that lymphadenectomy in patients with clinical stage pTI-IIb OCCC did not correlate with improved disease-free survival or overall survival (*P* = 0.353 and *P* = 0.645, respectively) [18].

While some studies have reported negative results [18–20, 32, 33, 38], it's important to exercise caution when interpreting these findings, as observational studies inherently come with limitations. In 2003, Ho et al. emphasized the potential benefits of complete surgical staging and the use of paclitaxel plus carboplatin for improved survival in stage I OCCC [39]. In 2011, a multicenter cooperative study involving 240 OCCC patients demonstrated that complete surgical staging, including lymphadenectomy, significantly improved disease-free survival (DFS) and overall survival (OS) [10]. However, a retrospective study based on the SEER database did not find a significant prolongation of survival in patients who underwent lymphadenectomy [20]. Nevertheless, there was a trend suggesting improved survival in patients with more than 10 lymph nodes removed and negative histology. This implies that more extensive lymphadenectomy may provide accurate staging and prognostic information. In another retrospective study involving advanced-stage patients from Japan, Kajiyama et al. could not establish the benefit of systematic retroperitoneal lymphadenectomy [33]. However, their findings did not refute the effectiveness of surgical resection for bulky, enlarged lymph nodes in achieving optimal cytoreduction.

Some authors and the International Federation of Gynecology and Obstetrics (FIGO) currently recommend complete surgical staging in early ovarian cancer [40–42]. This is because conventional histopathological examination may not detect certain micro-metastases, and comprehensive lymphadenectomy can potentially remove them [43, 44]. Previous studies have shown that up to 30% of women initially presumed to have early ovarian cancer are upstaged during restaging procedures, necessitating adjuvant therapy [45–47]. Accurate staging in very early disease can help avoid unnecessary postoperative chemotherapy. In advanced stages, lymphadenectomy serves a therapeutic purpose, striving to achieve optimal cytoreduction. Notably, compared to serous carcinoma, clear cell carcinoma has a higher frequency of lymph node metastasis upon recurrence [48], and lymph node involvement in clear cell carcinoma is associated with a poor prognosis [19, 37]. This underscores the potential benefit of systematic lymphadenectomy for patients with advanced OCCC, who face an increased risk of occult lymph node metastases. However, the elimination of occult metastases through lymphadenectomy remains a topic of debate, even though these metastases may contribute to recurrence. It's possible that isolated lymph node metastases may coexist with further micro-metastases that can spread to other lymph nodes or distant organs through numerous lymphatic vessels [33]. In cases where fertility preservation is a consideration, Takano et al. argued that lymphadenectomy should not be dismissed easily when diagnosing OCCC [49]. Gynecologic oncologists may face a dilemma, deciding whether to perform this surgery on all early cases or to spare a few patients with a poor prognosis from detection and treatment.

To the best of our knowledge, this is the first systematic review and meta-analysis examining the influence of lymphadenectomy on OCCC survival. However, our study has several limitations that warrant consideration. Firstly, some studies lacked information on intraoperative resection. Given OCCC's limited response to conventional chemotherapy, satisfactory cytoreductive surgery becomes particularly important. However, the absence of data on residual tumor status and peritoneal staging in certain studies, which are potential prognostic factors for OCCC patients, may have introduced bias into our results. Secondly, the quality of lymphadenectomy could not be assessed, and the number of lymph nodes removed varied among the included studies. This variability is a critical confounder that should be addressed in future research. Thirdly, there was no consistent definition of lymphadenectomy across the included studies. While some studies performed both pelvic and para-aortic lymphadenectomy, others focused on only one technique. This discrepancy could significantly bias the review's outcomes. Therefore, defining lymphadenectomy rigorously is crucial for accurate lymph node assessment. Fourthly, the combination of clear-cell histologic type with other ovarian cancer histologic types is not uncommon in clinical practice. Some OCCC studies did not exclude mixed cases, and few considered the distinction between pure and mixed clear cell carcinoma. This factor also had some impact on the results. Lastly, the effect of lymphadenectomy on survival remains uncertain due to the lack of well-designed RCT for OCCC. Therefore, it is premature to draw definitive conclusions regarding the impact of lymphadenectomy on OCCC patient survival. Conducting an RCT with an adequate number of OCCC cases is imperative to establish the significance of lymphadenectomy in OCCC.

## Conclusions

While lymphadenectomy can provide clinical benefits in certain cases of OCCC, it may not be universally applicable. We recommend that gynecologic oncologists consider developing tailored treatment strategies for high-risk subgroups, particularly OCCC patients at risk of lymph node metastasis. Efforts should be made to detect lymph node metastases both before and during surgery to identify those who stand to gain from lymphadenectomy. It's important to approach the divergent results observed in similar studies with caution. The true value of lymphadenectomy in the management of ovarian clear cell carcinoma warrants further investigation through large-scale prospective randomized controlled trials.

### Supplementary Information

**Additional file 1.** PRISMA checklist.

**Additional file 2.** Search Strategy.

**Additional file 3. **Full-text articles that were excluded (6) and the reason for exclusion.

**Additional file 4. **Risk of bias assessment of the included cohort studies.

## Data Availability

All data relevant to the study are included in the article or uploaded as supplementary information.

## Abbreviations

OCCC: Ovarian clear cell carcinoma
EOC: Epithelial ovarian cancer
HR: Hazard ratio
CI: Confidence intervals
DSS: Disease-specific survival
DFS: Disease-free survival
OS: Overall survival
PFS: Progression-free survival
NCCN: National Comprehensive Cancer Network
RCT: Randomized controlled trial
LION: Lymphadenectomy in Ovarian Neoplasm
PRISMA: Preferred Reporting Items for Systematic Reviews and Meta-Analysis
