# Supplementary material for: The impact of lymphadenectomy on ovarian clear cell carcinoma: a systematic review and meta-analysis
Source: World J Surg Oncol. 2024 Jan 29;22:37. doi: 10.1186/s12957-024-03324-6 (PMC10823682; doi:10.1186/s12957-024-03324-6)
Supplement: Supplementary file 1 — Additional file 1. PRISMA checklist. [file 12957_2024_3324_MOESM1_ESM.doc]

| **Section/topic** | **#** | **Checklist item** | **Reported on page #** |
| --- | --- | --- | --- |
| **TITLE** | | |  |
| Title | 1 | The report is identified as a systematic review and meta-analysis. | 1 |
| **ABSTRACT** | | |  |
| Structured summary | 2 | The structured abstract includes Objective, Methods, Results, Conclusion. | 2-3 |
| **INTRODUCTION** | | |  |
| Rationale | 3 | Described in the Introduction. | 4-6 |
| Objectives | 4 | Stated in the Introduction. | 6 |
| **METHODS** | | |  |
| Protocol and registration | 5 | The protocol is described in the Methods. The study was registered in the International Prospective Register of Systematic Reviews (PROSPERO; Register Number: CRD42021270460). | 6 |
| Eligibility criteria | 6 | Described in the Methods. | 6-7 |
| Information sources | 7 | Described in the Methods. | 6-7 |
| Search | 8 | The full search strategy is provided in Additional file 2. | Additional file 2 |
| Study selection | 9 | Described in the Methods. | 6-7 |
| Data collection process | 10 | Described in the Methods. | 7 |
| Data items | 11 | Described in the Methods. | 7 |
| Risk of bias in individual studies | 12 | Described in the Methods. | 7 |
| Summary measures | 13 | HR | 7 |
| Synthesis of results | 14 | Described in the Methods. | 7-8 |

Page 1 of 2

| **Section/topic** | **#** | **Checklist item** | **Reported on page #** |
| --- | --- | --- | --- |
| Risk of bias across studies | 15 | Described in the Methods. | 8 |
| Additional analyses | 16 | Described in the Methods. | 7-8 |
| **RESULTS** | | |  |
| Study selection | 17 | A flow-chart of study retrieval and selection is depicted in Fig.1 and reasons for full-text exclusions are given in additional file 3. | Fig.1  additional file 3 |
| Study characteristics | 18 | Details of included studies are presented in Table 1. | Table 1 |
| Risk of bias within studies | 19 | Detailed risk of bias assessment is described in additional file 4. | additional file 4 |
| Results of individual studies | 20 | Described in the Results. | 9 |
| Synthesis of results | 21 | Described in the Results. | 9-11 |
| Risk of bias across studies | 22 | Described in the Results. | 11 |
| Additional analysis | 23 | Described in the Results. | 9-11 |
| **DISCUSSION** | | |  |
| Summary of evidence | 24 | Described in the Discussion. | 11 |
| Limitations | 25 | Described in the Discussion. | 15-16 |
| Conclusions | 26 | Described in the Conclusion. | 16 |
| **FUNDING** | | |  |
| Funding | 27 | Described in the Funding. | 17 |

*From:*  Moher D, Liberati A, Tetzlaff J, Altman DG, The PRISMA Group (2009). Preferred Reporting Items for Systematic Reviews and Meta-Analyses: The PRISMA Statement. PLoS Med 6(7): e1000097. doi:10.1371/journal.pmed1000097

For more information, visit: **www.prisma-statement.org**.

Page 2 of 2
