# Supplementary material for: The impact of lymphadenectomy on ovarian clear cell carcinoma: a systematic review and meta-analysis
Source: World J Surg Oncol. 2024 Jan 29;22:37. doi: 10.1186/s12957-024-03324-6 (PMC10823682; doi:10.1186/s12957-024-03324-6)
Supplement: Supplementary file 2 — Additional file 2. Search Strategy. [file 12957_2024_3324_MOESM2_ESM.doc]

**Additional file 2. Search Strategy**

1. **PubMed**

**Concept 1 clear cell carcinoma**

("Adenocarcinoma, Clear Cell"[Mesh]) OR (((Adenocarcinomas, Clear Cell [Title/Abstract]) OR (Clear Cell Adenocarcinoma [Title/Abstract])) OR (Clear Cell Adenocarcinomas [Title/Abstract]))

**Concept 2 ovarian cancer**

(((((((((((((((((Neoplasm, Ovarian[Title/Abstract]) OR (Ovarian Neoplasm[Title/Abstract])) OR (Ovary Neoplasms[Title/Abstract])) OR (Neoplasm, Ovary[Title/Abstract])) OR (Neoplasms, Ovary[Title/Abstract])) OR (Ovary Neoplasm[Title/Abstract])) OR (Neoplasms, Ovarian[Title/Abstract])) OR (Ovary Cancer[Title/Abstract])) OR (Cancer, Ovary[Title/Abstract])) OR (Cancers, Ovary[Title/Abstract])) OR (Ovary Cancers[Title/Abstract])) OR (Ovarian Cancer[Title/Abstract])) OR (Cancer, Ovarian[Title/Abstract])) OR (Cancers, Ovarian[Title/Abstract])) OR (Ovarian Cancers[Title/Abstract])) OR (Cancer of Ovary[Title/Abstract])) OR (Cancer of the Ovary[Title/Abstract])) OR ("Ovarian Neoplasms"[Mesh])

**Concept 3 lymphadenectomy**

(((((((((((Excision, Lymph Node[Title/Abstract]) OR (Excisions, Lymph Node[Title/Abstract])) OR (Lymph Node Excisions[Title/Abstract])) OR (Lymphadenectomy[Title/Abstract])) OR (Lymphadenectomies[Title/Abstract])) OR (Lymph Node Dissection [Title/Abstract])) OR (Dissection, Lymph Node[Title/Abstract])) OR (Dissections, Lymph Node[Title/Abstract])) OR (Lymph Node Dissections[Title/Abstract])) OR (Node Dissection, Lymph[Title/Abstract])) OR (Node Dissections, Lymph[Title/Abstract])) OR ("Lymph Node Excision"[Mesh])

**Concept 1 AND Concept 2 AND Concept 3**

1. **Web of science**

**Concept 1 clear cell carcinoma**

TS=(Adenocarcinoma, Clear Cell OR Adenocarcinomas, Clear Cell OR Clear Cell Adenocarcinoma OR Clear Cell Adenocarcinomas)

**Concept 2 ovarian cancer**

TS=(Neoplasm, Ovarian OR Ovarian Neoplasm OR Ovary Neoplasms OR Neoplasm, Ovary OR Neoplasms, Ovary OR Ovary Neoplasm OR Neoplasms, Ovarian OR Ovary Cancer OR Cancer, Ovary OR Cancers, Ovary OR Ovary Cancers OR Ovarian Cancer OR Cancer, Ovarian OR Cancers, Ovarian OR Ovarian Cancers OR Cancer of Ovary OR Cancer of the Ovary OR Ovarian Neoplasms)

**Concept 3 lymphadenectomy**

TS=(Excision, Lymph Node OR Excisions, Lymph Node OR Lymph Node Excisions OR Lymphadenectomy OR Lymphadenectomies OR Lymph Node Dissection OR Dissection, Lymph Node OR Dissections, Lymph Node OR Lymph Node Dissections OR Node Dissection, Lymph OR Node Dissections, Lymph OR Lymph Node Excision)

**Concept 1 AND Concept 2 AND Concept 3**

1. **Scopus**

**Concept 1 clear cell carcinoma**

TITLE-ABS-KEY (adenocarcinomas, AND clear AND cell) OR TITLE-ABS-KEY (clear AND cell AND adenocarcinoma) OR TITLE-ABS-KEY (clear AND cell AND adenocarcinomas) OR TITLE-ABS-KEY (adenocarcinoma, AND clear AND cell)

**Concept 2 ovarian cancer**

TITLE-ABS-KEY (neoplasm, AND ovarian) OR TITLE-ABS-KEY (ovarian AND neoplasm) OR TITLE-ABS-KEY (ovary AND neoplasms) OR TITLE-ABS-KEY (neoplasm, AND ovary) OR TITLE-ABS-KEY (neoplasms, AND ovary) OR TITLE-ABS-KEY (ovary AND neoplasm) OR TITLE-ABS-KEY (neoplasms, AND ovarian) OR TITLE-ABS-KEY (ovary AND cancer) OR TITLE-ABS-KEY (cancer, AND ovary) OR TITLE-ABS-KEY (cancers, AND ovary) OR TITLE-ABS-KEY (ovary AND cancers) OR TITLE-ABS-KEY (ovarian AND cancer) OR TITLE-ABS-KEY (cancer, AND ovarian) OR TITLE-ABS-KEY (cancers, AND ovarian) OR TITLE-ABS-KEY (ovarian AND cancers ) OR TITLE-ABS-KEY (cancer AND of AND ovary) OR TITLE-ABS-KEY (cancer AND of AND the AND ovary) OR TITLE-ABS-KEY (ovarian AND neoplasms)

**Concept 3 lymphadenectomy**

TITLE-ABS-KEY (lymph AND node AND excision) OR TITLE-ABS-KEY (excision, AND lymph AND node) OR TITLE-ABS-KEY (excisions, AND lymph AND node) OR TITLE-ABS-KEY (lymph AND node AND excisions) OR TITLE-ABS-KEY (lymphadenectomy) OR TITLE-ABS-KEY (lymphadenectomies) OR TITLE-ABS-KEY (lymph AND node AND dissection) OR TITLE-ABS-KEY (dissection, AND lymph AND node ) OR TITLE-ABS-KEY (dissections, AND lymph AND node) OR TITLE-ABS-KEY (lymph AND node AND dissections) OR TITLE-ABS-KEY (node AND dissection, AND lymph) OR TITLE-ABS-KEY (node AND dissections, AND lymph)

**Concept 1 AND Concept 2 AND Concept 3**
