# Supplementary material for: The impact of lymphadenectomy on ovarian clear cell carcinoma: a systematic review and meta-analysis
Source: World J Surg Oncol. 2024 Jan 29;22:37. doi: 10.1186/s12957-024-03324-6 (PMC10823682; doi:10.1186/s12957-024-03324-6)
Supplement: Supplementary file 3 — Additional file 3. Full-text articles that were excluded (6) and the reason for exclusion. [file 12957_2024_3324_MOESM3_ESM.docx]

**Additional file 3. Full-text articles that were excluded (6) and the reason for exclusion**.

For each study the first author, country, year of publication and type of study is displayed.

| First author | Country | Year | Reason for exclusion | Study type |
| --- | --- | --- | --- | --- |
| Chih-Ming Ho | Taiwan | 2003 | Insufficient patients included | Observational |
| John K. Chan | USA | 2007 | Unable to extract data | Observational |
| Masashi Takano | Japan | 2008 | Insufficient patients included | Observational |
| Akiko Abe | Japan | 2010 | Insufficient patients included | Observational |
| Kayo Suzuki | Japan | 2014 | Unable to extract data | Observational |
| Koji Matsuo | USA | 2018 | Lack of valid data | Observational |
