# Supplementary material for: The impact of lymphadenectomy on ovarian clear cell carcinoma: a systematic review and meta-analysis
Source: World J Surg Oncol. 2024 Jan 29;22:37. doi: 10.1186/s12957-024-03324-6 (PMC10823682; doi:10.1186/s12957-024-03324-6)
Supplement: Supplementary file 4 — Additional file 4. Risk of bias assessment of the included cohort studies. [file 12957_2024_3324_MOESM4_ESM.doc]

**Additional file 4. Risk of bias assessment of the included cohort studies.**

| Study, year | Selection | | | | Comparability | outcome | | | Total score |
| --- | --- | --- | --- | --- | --- | --- | --- | --- | --- |
|  | Exposed cohort | Non-exposed cohort | Ascertainment of exposure | Outcome of interest |  | Assessment of outcome | Length of follow-up | Adequacy of follow up |  |
| Suzuki at el. 2008 | ☆ | ☆ | ☆ | - | ☆☆ | ☆ | ☆ | ☆ | 8 |
| Takano at el.2009 | ☆ | ☆ | ☆ | - | ☆☆ | ☆ | ☆ | ☆ | 8 |
| Magazzino at el. 2011 | ☆ | ☆ | ☆ | - | ☆ | ☆ | ☆ | ☆ | 7 |
| Mahdi at el. 2013 | ☆ | ☆ | ☆ | - | ☆☆ | ☆ | ☆ | ☆ | 8 |
| Yamazaki at el. 2018 | ☆ | ☆ | ☆ | - | ☆☆ | ☆ | ☆ | ☆ | 8 |
| Kajiyama at el. 2020 | ☆ | ☆ | ☆ | - | ☆☆ | ☆ | ☆ | ☆ | 8 |
| Nasioudis at el. 2021 | ☆ | ☆ | ☆ | - | ☆ | ☆ | ☆ | ☆ | 7 |

Risk of bias was evaluated with use of the Newcastle-Ottawa Scale. A score of 7 or higher indicates a low risk of bias.
